# A new silicon tracker for proton imaging and dosimetry

**DOI:** 10.1016/j.nima.2016.02.013

**Published:** 2016-09-21

**Authors:** J.T. Taylor, C. Waltham, T. Price, N.M. Allinson, P.P. Allport, G.L. Casse, A. Kacperek, S. Manger, N.A. Smith, I. Tsurin

**Affiliations:** a Department of Physics, University of Liverpool, Oxford Street, Liverpool L69 7ZE, UK; b Laboratory of Vision Engineering, School of Computer Science, University of Lincoln, Lincoln LN6 7TS, UK; c School of Physics and Astronomy, University of Birmingham, Birmingham B25 2TT, UK; d Douglas Cyclotron, The Clatterbridge Cancer Centre NHS Foundation Trust, Clatterbridge Road, Bebington, Wirral CH63 4JY, UK; e Department of Physics, University of Warwick, Coventry CV4 7AL, UK

**Keywords:** Silicon tracking detectors, Proton therapy, Dosimetry, Proton computed tomography

## Abstract

For many years, silicon micro-strip detectors have been successfully used as tracking detectors for particle and nuclear physics experiments. A new application of this technology is to the field of particle therapy where radiotherapy is carried out by use of charged particles such as protons or carbon ions. Such a treatment has been shown to have advantages over standard x-ray radiotherapy and as a result of this, many new centres offering particle therapy are currently under construction around the world today. The Proton Radiotherapy, Verification and Dosimetry Applications (PRaVDA) consortium are developing instrumentation for particle therapy based upon technology from high-energy physics.

The characteristics of a new silicon micro-strip tracker for particle therapy will be presented. The array uses specifically designed, large area sensors with technology choices that follow closely those taken for the ATLAS experiment at the HL-LHC. These detectors will be arranged into four units each with three layers in an *x*–*u*–*v* configuration to be suitable for fast proton tracking with minimal ambiguities.

The sensors will form a tracker capable of tracing the path of ~200 MeV protons entering and exiting a patient allowing a new mode of imaging known as proton computed tomography (*p*CT). This will aid the accurate delivery of treatment doses and in addition, the tracker will also be used to monitor the beam profile and total dose delivered during the high fluences used for treatment.

We present here details of the design, construction and assembly of one of the four units that will make up the complete tracker along with its characterisation using radiation tests carried out using a ^90^Sr source in the laboratory and a 60 MeV proton beam at the Clatterbridge Cancer Centre.

## 1 Introduction

*Particle therapy*: alongside surgery and chemotherapy, radiotherapy remains one of the three major tools used by clinicians to combat cancer today. Typically, the standard approach for radiotherapy involves the use of x-ray beams, a technique that was first developed for medicine by physicists. Radiotherapy today, still remains a field that continues to go hand in hand with developments in physics and engineering [1]. For cancers deep inside the body or close to critical structures, particle therapy has been shown to have a distinct advantage over standard x-ray radiotherapy. This is a result of the underlying physics that describes how radiation interacts with matter. For charged particles, such as protons, energy loss is described by the well established Bethe–Bloch formula [2] and results in a characteristic ‘Bragg Peak’. This is where the majority of the particles stop and where their dE/dx or Linear Energy Transfer (LET) is maximal, resulting in a concentration of the dose in this region. Taking advantage of this dose distribution for radiotherapy was first envisaged by Wilson [3]. For x-rays, energy is lost in an exponential fashion and thus much of the dose is given to healthy tissue before and after the tumour. This can be avoided by using a beam of charged particles such as protons. This sparing of healthy tissue also makes particle therapy the best choice for some childhood cancers, since growing tissues and bones are more radiosensitive than in adults and critical organs are located much closer together. In this case, the excess dose to healthy tissue can cause irreparable damage to developing cells that can cause other cancers later on in life [4]. For this reason, many of the patients that the National Health Service (NHS) in the UK currently sends abroad each year for proton therapy treatment are children [5]. Because of these advantages, approximately 50 centres offering particle therapy have been built around the world, with many new centres under construction worldwide including two new NHS proton facilities in the UK.

**Fig. 1:**
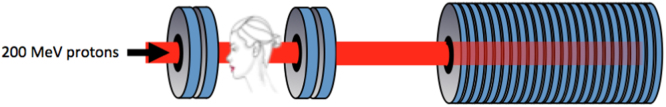
The PRaVDA *p*CT system concept. The tracker is comprised of the first four units shown here as the two which are placed in front of the object to be imaged and the two after. A range telescope (calorimeter) is placed immediately after the tracker to measure the residual energy of each proton after it has been tracked through the object to be imaged.

*Proton imaging*: instrumental to the planning of any program of radiotherapy is a good imaging modality that can deliver accurate information on the patient׳s anatomy, and in particular the accurate location of the target volume. For proton therapy, this is carried out by an x-ray CT scan from which the proton stopping power of the tissue can be derived and the necessary range of the treatment beam calculated. During this conversion from x-ray imaging to proton stopping power, an uncertainty in the proton range is introduced, of order 1–3 mm [6]. This arises from variations in density along the proton path [7] and inaccuracies in the excitation energy or electron density value assumed for the tissue [8], as well as from the stopping power conversion method itself [9]. Uncertainties in the proton range increase the amount of dose delivered to the healthy tissue surrounding the cancer and can therefore prevent the treatment of cancers close to critical structures. If this uncertainty could be reduced, several of the limitations with proton therapy could be overcome. These uncertainties could potentially be reduced if the stopping power of the beam could be measured directly i.e. by using protons for imaging as well as for treatment [10]. In order to carry out this technique (referred to as proton computed tomography, or simply *p*CT), a device that can accurately measure the trajectory and energy loss of protons as they pass through an object for many different angular projections is needed [11], [12], [13], [14]. The PRaVDA Consortium [15] aims to construct a prototype of the first fully solid state *p*CT scanner using silicon detectors for both the tracking and range (energy) measurements of protons (see Fig. 1) [16], [17], [18], [19]. We have designed and begun construction of the silicon strip tracker, using large area (~10×10 cm) micro-strip detectors adapted from designs made for the ATLAS experiment at the high-luminosity LHC allowing the sensors to be very radiation hard [20], [21]. The sensitive area of the detectors was constrained by the available space on a standard 6-in. silicon wafer and when arranged in the proposed *x*–*u*–*v* configuration provides an imaging area of ~9 cm. Future designs for imaging larger, more clinically relevant areas could be done by using these detectors ganged together (with some dead regions), or by moving the imaging system itself. The current design of the tracker will demonstrate that a completely solid state system offering high-precision directional information on the path of protons can be used in conjunction with an energy-range measurement to perform a *p*CT scan [22].

## 2 Assembly and readout of the tracking units

**Fig. 2:**
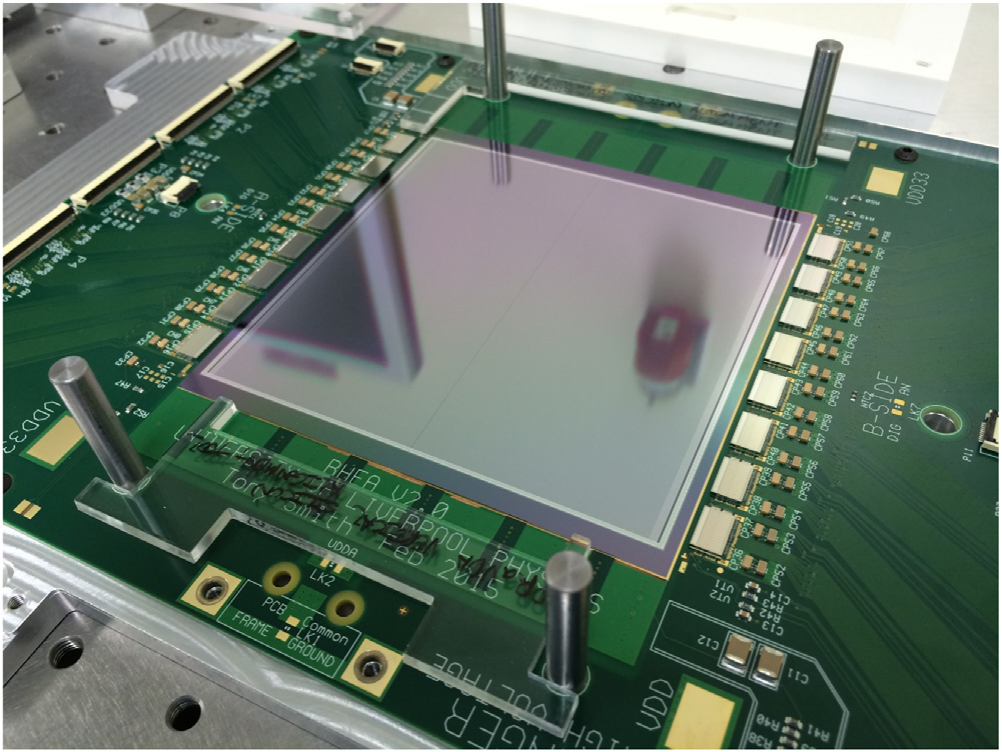
Alignment of detector to hybrid PCB and aluminium stiffener plate using precision ground tooling consisting of dowels and perspex jigs.

*Silicon micro-strip sensor*: each silicon micro-strip detector has a nominal thickness of 150 μm and is made from n-in-p silicon. The detector contains 2048 strips in total, 1024 read out on each side of the detector by eight ASICs (see Fig. 2). Each strip has a pitch of 90.8 μm and a length of 4.8 cm and its metal layer is capacitively coupled to its implant with a measured coupling capacitance of 122 pF. Further details of the layout and electrical characteristics of the sensor can be found here [22].

Detectors are aligned to the hybrid PCB and a 12 mm thick aluminium stiffener plate that holds the detector and its associated readout electronics within the tracker unit housing (see Fig. 4). Both the PCB and the aluminium plate contain a square 10×10 cm aperture beneath the sensitive area of the detector to keep the perturbation of the proton path to a minimum. Initial mechanical alignment of the sensors is made using precision ground dowels and a custom built alignment jig with precision ground edges (see Fig. 2) and carried out as part of the gluing and assembly process for each detector used. The alignment achieved with this tooling was found to be within the strip pitch of the detector (90.8 μm) and the rotational alignment to be a few mrad. This was measured using a Smartscope metrology machine which makes optical measurements using a camera in order to estimate the height and flatness and lateral position of the silicon above the PCB surface since the detector could not be probed mechanically due to its fragile nature. Further precision in alignment will be achievable using particle tracks when multiple tracking units are available.

The tracker is comprised of 12 detectors separated into four units each containing three detectors each. Two units are placed either side of the object to be imaged as shown in Fig. 1. Each tracking unit has its detectors held at an angle of 60° with respect to one another in an *x*–*u*–*v* co-ordinate system in order to measure precise *x*–*y* locations for particles at high fluence with minimal ambiguities. The angle is achieved by suspending the aluminium plates containing the micro-strip detectors and their associated readout electronics on six precision ground dowels (see Fig. 4).

**Fig. 3:**
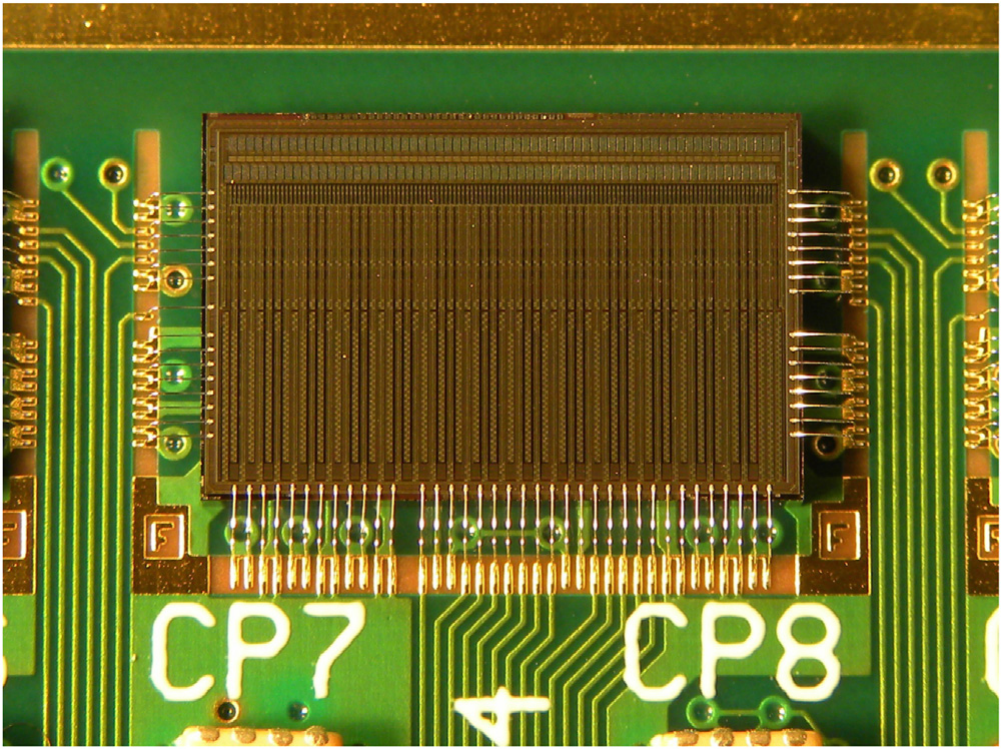
A RHEA ASIC measuring 8.2×5.2×0.85 mm with back end bonding to hybrid PCB. The staggered front-end bond pads that are empty are ready for wire-bonding to a micro-strip detector after gluing and assembly of the detector on to the hybrid PCB.

**Fig. 4:**
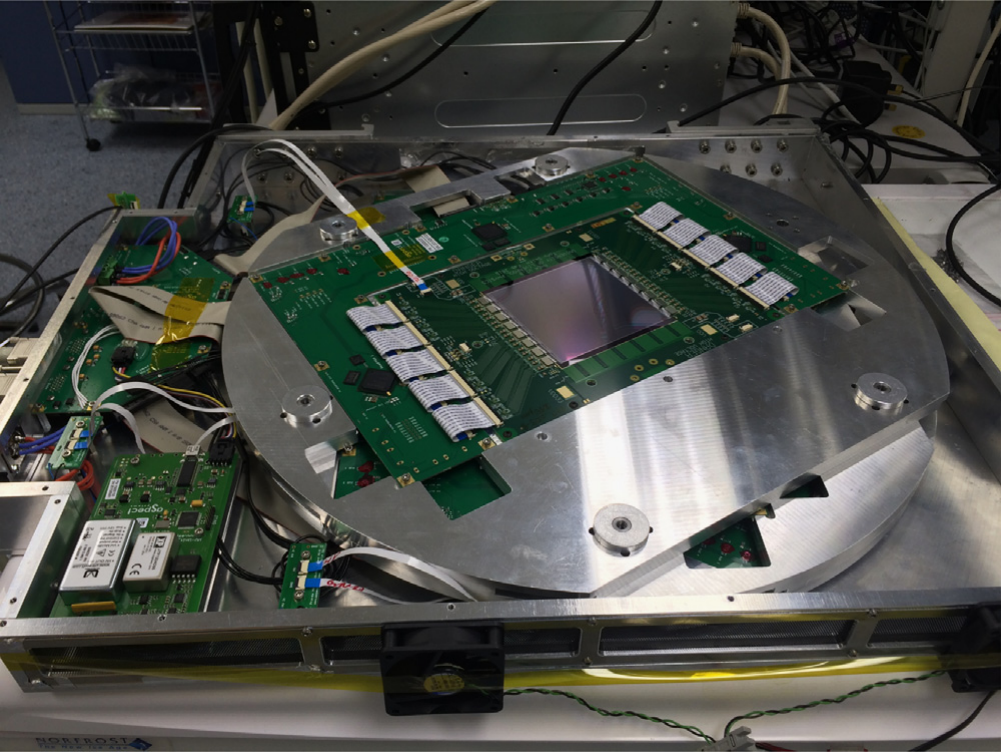
Setup of the three layer tracking unit assembled in the housing with a multiplexer board, HV unit and associated cables. The three layers that make up the tracking unit are secured using six dowels that provide registration and alignment at the required angle.

*Readout electronics*: the detector is read out by means of an ASIC designed specifically for this application by ISDI Ltd. [23] and known as RHEA (Rapid, High-speed Extended ASIC). RHEA is a binary chip with 128 channels and a bonding pitch of 60 μm fabricated in 0.18 μm CMOS (see Fig. 3). Each channel has two tunable thresholds (DAC1: 2000–10,000 e^−^, and DAC2: 20,000 – 160,000 e^−^) to allow for high occupancy, and the chip operates at a frequency of 104 MHz and its front-end amplifier with a shaping time of 30 ns. This corresponds to four times the average cyclotron frequency for the energy range of interest (60–200 MeV) at the facilities that will be used. There are two modes that can be used to acquire data: treatment mode and patient imaging or *p*CT mode. In treatment mode all strips are read out at ~100 μs intervals to allow sampling of the beam distribution for quality assurance (QA) purposes and dosimetry during the high fluences used during patient treatment. In *p*CT mode, it is possible to read up to four channels per ASIC with signal over threshold for the expected nominal beam spill repetition rate of 26 MHz. This allows for accurate tracking of multiple protons through the system at any one time. The hybrid PCB which can be seen in Fig. 2 is used to mount the detector and RHEA chips. It is responsible for delivering power, calibration and serial interface data to the ASICs as well as routing the data output from the ASICs to the FPGA׳s that are mounted on a data acquisition (DAQ) board which surrounds the hybrid (see Fig. 4). The DAQ boards and associated software were designed by aSpect Systems GmbH [24].

## 3 Experimental work and results

**Fig. 5:**
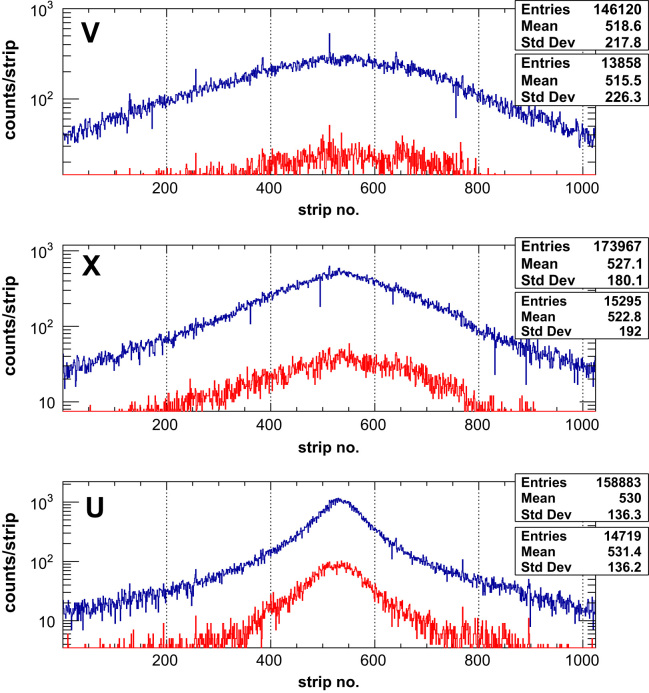
Distribution of MIPs from a ^90^Sr source measured in each of the three layers of the tracking unit. The upper histogram in each frame (blue data) is for the first threshold and the lower histogram in each frame (red data) for the second threshold. The top layer closest to the source is labelled as ‘U’ and is orientated at +60°. The middle layer labelled ‘X’ is orientated at 0°, and the bottom layer labelled ‘V’ is orientated at −60°. (For interpretation of the references to colour in this figure caption, the reader is referred to the web version of this paper.)

*Tests with minimum ionising particles*: preliminary tests with the assembled tracking unit were carried out in the lab using minimum ionising particles (MIPs) from a ^90^Sr source with a activity of 37 MBq. Although the tracker was never designed to track such particles, it served as a useful diagnostic during assembly to see, for each layer, if the signals were visible above the noise and could be read out with the DAQ. This test was repeated after construction of the tracking unit in order to verify that signals could be seen separately in each of the three layers (and six strip halves) in order to optimise the spacing of the layers and prevent crosstalk. Such tests provided valuable information for adjustments of the electronics and improvement of the DAQ software, as well as allowing sensor/ASIC characteristics to be extracted during times when tests at an accelerator were not possible. Using these methods, the number of dead or noisy strips turned off by the DAQ was found on average to be <0.5% per layer. The most probable value for the expected signal size of MIPs in the 150 μm thick detectors used is: ~12,000 e^−^. This signal is less than half of what will be expected in the final application when beams of protons with energies ranging from 60 to 200 MeV are used and thus, optimisation of the system for detection of MIPs allowed the system to be prepared for tests in a proton beam. In Fig. 5, the 1D hit maps for data from the ^90^Sr source is shown in the three layers of a tracking unit formed from the data of six strip halves. This data was collected for several thousand frames in the treatment mode readout (which reads out all available channels) and allowed the testing of all channels in both the first and second threshold. This can be seen in the blue and red histograms respectively, set at their default values of 84 and 200 mV. which correspond to the end of the first threshold (~10,000 e^−^ and the middle of the second threshold (~90,000 $e^{-}$).

**Fig. 6:**
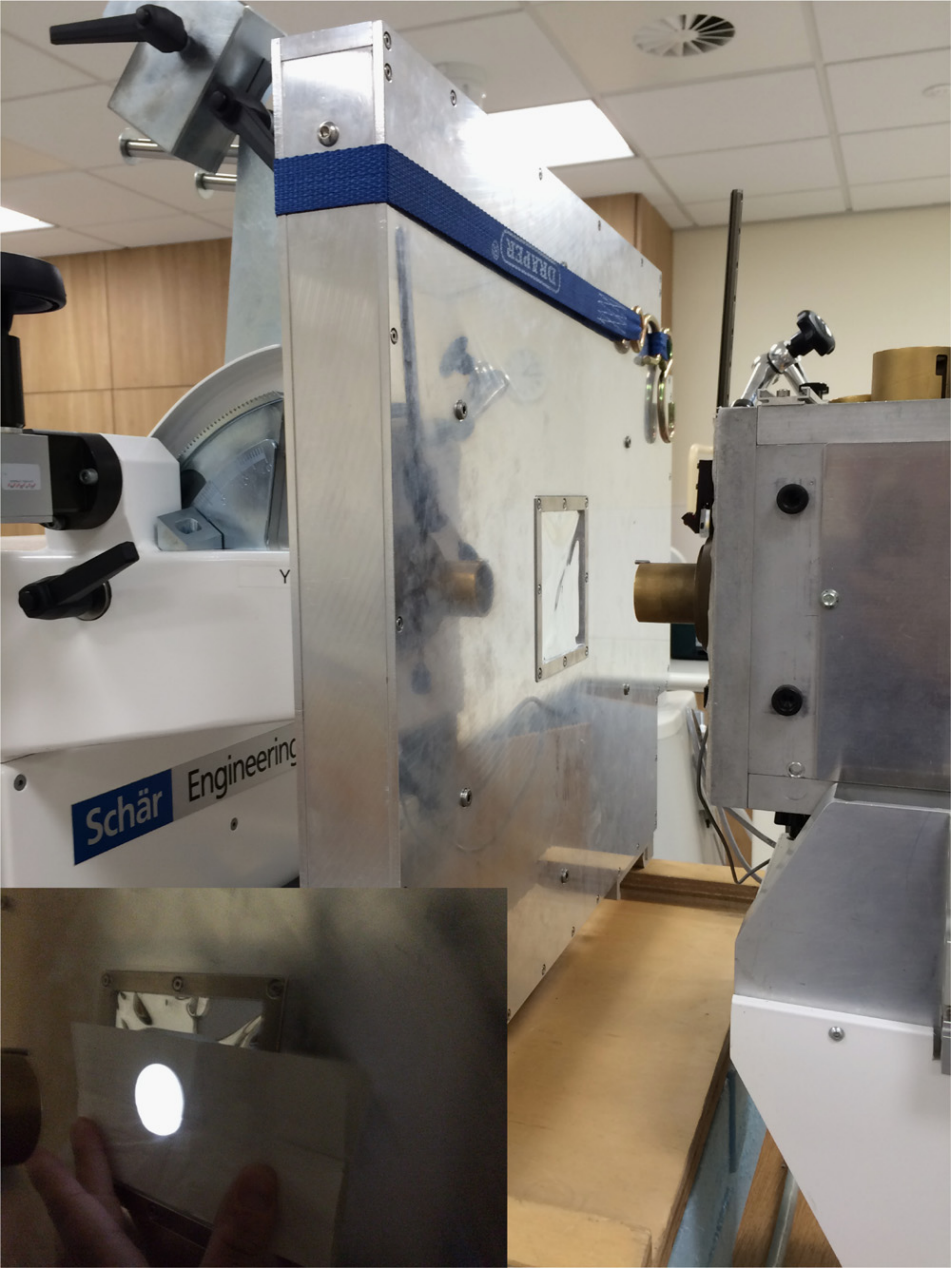
Experimental setup at the Clatterbridge Cancer Centre. The inset figure shows alignment of the tracking unit׳s sensitive area with the beam using the field lamp.

*Tests with a proton beam*: tests were carried out using a 60 MeV beam of protons from the Douglas Cyclotron at the Clatterbridge Cancer Centre. This is currently the only clinical proton beam in the UK and has been used for research as well as for the treatment of eye cancers for many years [25]. Fig. 6 shows the setup on the Clatterbridge beam line with the single tracking unit positioned at the end of the treatment nozzle. A ‘field lamp’ (shown in the inset of Fig. 6) was used to check for reasonable alignment of the active area of the sensor with the beam emerging from the nozzle.

**Fig. 7:**
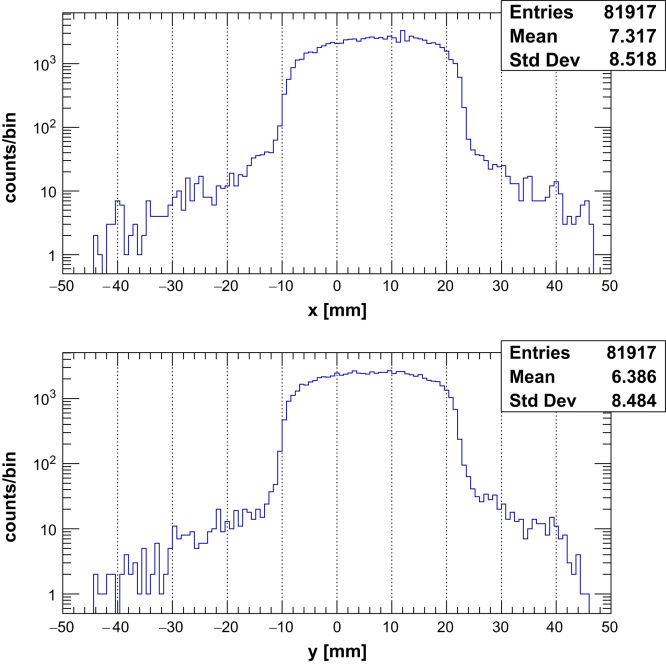
The 1D reconstructed hit maps in *x* and *y* of a 30 mm, 60 MeV proton beam at the Clatterbridge Cancer Centre. The distributions were reconstructed into 800 μm bins using two planes of the tracker unit orientated at 60° to one another.

**Fig. 8:**
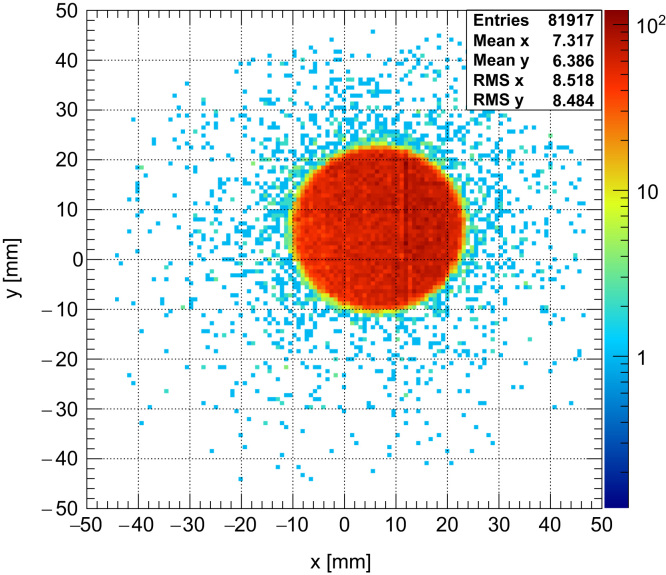
The 2D reconstructed hit map of a 30 mm, 60 MeV proton beam at the Clatterbridge Cancer Centre. The distribution was reconstructed into 800 μm bins using two planes of the tracker unit orientated at 60° to one another.

During the experiment, a beam of protons with a width of 30 mm was used and its fluence monitored by means of an ionisation chamber. The chamber (connected to an electrometer) was situated a few cm after the nozzle in front of the detector (not shown in Fig. 6) and the background current observed when the beam was off was found to be <1pA. A typical current of between 5 and 10 pA was used for all measurements which ensured that each successive frame would contain on average only one proton in order to be sure of characterising the system without any pile-up or saturation present. The reconstructed (*x*,*y*) locations of the protons for the 30 mm beam at a beam current of 7 pA can be seen for data in the first threshold in Fig. 7. The effect of the scattering foil placed upstream of the detectors is evidenced by the flat top of the distributions. Data was collected for 1 s using the CT mode where each successive event receives a timestamp depending on the time it occurs relative to the internal clock supplied to the ASIC by the FPGA on the DAQ readout board. This allowed the proton hits in the unit to be tracked across subsequent layers and reconstructed (*x*,*y*) locations calculated. A two-dimensional hit map of the beam using these locations can be seen in Fig. 8 reconstructed using two layers in the tracking unit. The addition of more layers and tracking units read out in this mode will be used in order to carry out a *p*CT scan.

## 4 Conclusion

We have presented here the assembly and first results of a three layer tracking unit that will be deployed along with three other units for particle therapy applications. The results presented here show that both modes of data acquisition and both thresholds are operational and that tracking between the layers is possible. The complete tracker will be capable of fast particle tracking with minimal ambiguities and can therefore give useful measurements for dosimetry and beam QA, as well as providing the necessary directional information on the path of individual protons during a *p*CT scan for proton imaging. Future papers will concentrate on the reconstruction of 2D images from all layers across multiple tracking units, as well as the tracking of protons between units, in order to measure the scattering angles for a particular beam configuration. Furthermore, the ability of the tracker to track through a thick target (~75 mm phantom) in conjunction with an energy-range measurement of the protons will also be assessed. This is necessary in order to know whether it will be possible to demonstrate that a *p*CT scan is possible with a fully solid state system of this kind.
